# Supplementary material for: The interactome of intact mitochondria by cross-linking mass spectrometry provides evidence for coexisting respiratory supercomplexes
Source: Mol Cell Proteomics. 2017 Dec 8;17(2):216–32. doi: 10.1074/mcp.RA117.000470 (PMC5795388; doi:10.1074/mcp.RA117.000470)
Supplement: Supplemental Data [file supp_RA117.000470_133922_0_supp_23978_fzffwf.zip › spectra_annotation/mito_DR_spectra_annotation/376-1-1-1-7-1.pdf]

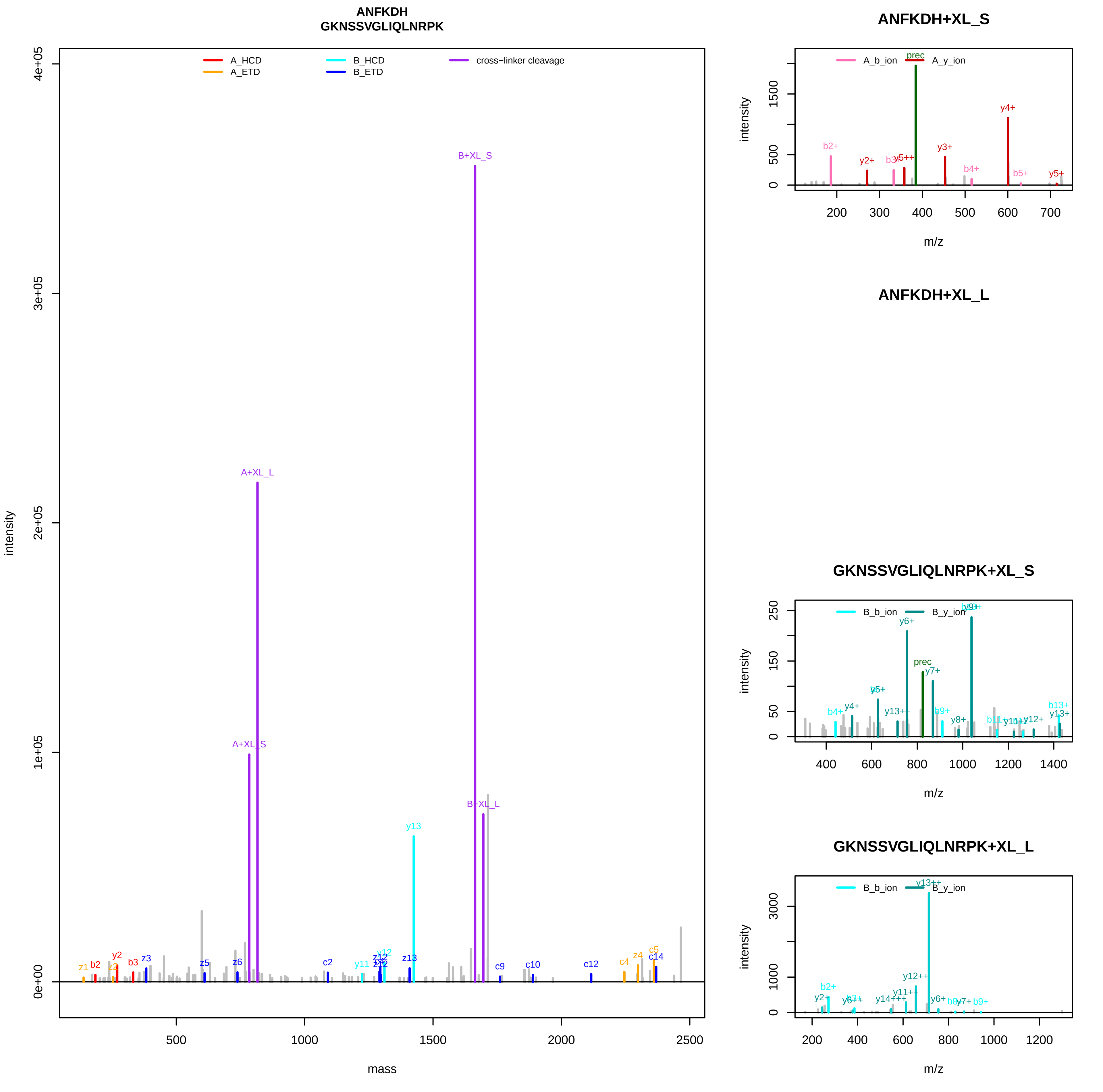
ANFKDH
GKNSSVGLIQLNRPK
A_HCD
A_ETD
B_HCD
B_ETD
cross−linker cleavage
B+XL_S
A+XL_L
A+XL_S
B+XL_L
y13
z1
b2
y2
b3
z3
z5
z6
c2
y11
y12
z12
z13
c9
c10
c12
c4
z4
c5
c14
intensity
mass
0e+00
1e+05
2e+05
3e+05
4e+05
500
1000
1500
2000
2500
ANFKDH+XL_S
A_b_ion
A_y_ion
prec
b2+
y2+
b3+
y5++
y3+
y4+
b4+
b5+
y5+
m/z
ANFKDH+XL_L
GKNSSVGLIQLNRPK+XL_S
B_b_ion
B_y_ion
y6+
prec
y7+
y5+
b4+
y4+
y13+
b9+
y8+
y11++
y12+
b13+
y13+
GKNSSVGLIQLNRPK+XL_L
y13++
b2+
y2+
y6++
y12++
y11++
y14+++
y6+
y7+
b9+
